# Compatibility of High-Moisture Storage for Biochemical Conversion of Corn Stover: Storage Performance at Laboratory and Field Scales

**DOI:** 10.3389/fbioe.2018.00030

**Published:** 2018-03-26

**Authors:** Lynn M. Wendt, J. Austin Murphy, William A. Smith, Thomas Robb, David W. Reed, Allison E. Ray, Ling Liang, Qian He, Ning Sun, Amber N. Hoover, Quang A. Nguyen

**Affiliations:** ^1^Idaho National Laboratory, Idaho Falls, ID, United States; ^2^Independent Researcher, Olathe, KS, United States; ^3^Advanced Biofuels Process Development Unit, Lawrence Berkeley National Laboratory, Emeryville, CA, United States

**Keywords:** Corn stover, biomass storage, ensiling, Ritter pile, field storage, feedstock reactivity

## Abstract

Wet anaerobic storage of corn stover can provide a year-round supply of feedstock to biorefineries meanwhile serving an active management approach to reduce the risks associated with fire loss and microbial degradation. Wet logistics systems employ particle size reduction early in the supply chain through field-chopping which removes the dependency on drying corn stover prior to baling, expands the harvest window, and diminishes the biorefinery size reduction requirements. Over two harvest years, in-field forage chopping was capable of reducing over 60% of the corn stover to a particle size of 6 mm or less. Aerobic and anaerobic storage methods were evaluated for wet corn stover in 100 L laboratory reactors. Of the methods evaluated, traditional ensiling resulted in <6% total solid dry matter loss (DML), about five times less than the aerobic storage process and slightly less than half that of the anaerobic modified-Ritter pile method. To further demonstrate the effectiveness of the anaerobic storage, a field demonstration was completed with 272 dry tonnes of corn stover; DML averaged <5% after 6 months. Assessment of sugar release as a result of dilute acid or dilute alkaline pretreatment and subsequent enzymatic hydrolysis suggested that when anaerobic conditions were maintained in storage, sugar release was either similar to or greater than as-harvested material depending on the pretreatment chemistry used. This study demonstrates that wet logistics systems offer practical benefits for commercial corn stover supply, including particle size reduction during harvest, stability in storage, and compatibility with biochemical conversion of carbohydrates for biofuel production. Evaluation of the operational efficiencies and costs is suggested to quantify the potential benefits of a fully-wet biomass supply system to a commercial biorefinery.

## Introduction

Biomass is recognized as a renewable and sustainable energy resource, and presently 130 million tonnes of agricultural residues are available in the U.S. with the potential to supply up to 180 million dry tonnes of biomass for conversion to bioenergy by 2040 (Langholtz et al., 2016). In addition, the use of agricultural residues and other waste products to produce energy is being explored throughout the world as a means to provide income to rural communities (Dale et al., 2016; Nizami et al., 2017). However, because agricultural residue harvest is seasonal, long-term storage methods are needed to continuously supply feedstock throughout the year at a profitable refinery. Multiple strategies exist for designing the most cost-effective herbaceous feedstock logistics supply chains (Hess et al., 2007; Kumar and Singh, 2017; Shah et al., 2017; Zandi Atashbar et al., 2017), yet common to all of these is the reliance on dry bales for long-term storage. Although dry bales may have low dry matter loss (DML) in storage (<10%) when moisture content is below 20% wet basis (wb), they represent a significant source of combustible material and a fire risk (Rentizelas et al., 2009). Storage-related fires can lead to the losses of thousands of tons of biomass, may contribute to local air and water quality concerns, and in some cases may prompt legal action by adjacent property owners (Dale, 2017). In addition, microbial degradation of biomass, leading to DML, is also a significant risk if the bales are processed under conditions too wet for proper dry storage (over 25% moisture, wb) or if the bales absorb excessive moisture during storage (Kenney et al., 2013; Smith et al., 2013). DMLs that occur during outdoor storage of bales have been shown to be a significant contributor to delivered feedstock costs (Sahoo and Mani, 2017).

Corn stover is one of the primary agricultural residues available for producing bioenergy, but there are challenges associated with dry storage as a result of available harvest techniques. For example, since the final moisture content of the stover is fixed by the timing of the grain harvest, it is often higher than optimal for dry storage, especially in northern latitudes (Shinners and Binversie, 2007). In geographical regions where double-cropping is practiced, stover is removed soon after harvest to facilitate winter crop planting, which limits the time available for drying (Heggenstaller et al., 2008). In a multi-pass corn stover harvest, the stover is left in the field to dry to a moisture content of about 15–20% (wb) prior to baling (Darr and Shah, 2012); however, sufficient drying time is not always possible due to regional seasonal rain (Shah et al., 2011; Smith et al., 2013; Oyedeji et al., 2017). Oyedeji et al. (2017) estimated that less than 40% of nationally available corn stover meets the 20% moisture threshold at the time of baling. Likewise, single-pass harvesting is not compatible with field drying because it is baled at the same time as the grain is harvested (Shinners et al., 2011), although this approach has the advantage of reducing soil contamination by preventing stover contact with the ground.

Wet anaerobic storage (i.e., ensiling) is an attractive alternative to dry bale storage and can protect the biomass from biologically-mediated DML while reducing the risk of catastrophic fire loss. Wet storage through ensiling commonly utilizes silage bags, bunkers, or drive-over piles for oxygen limitation availability, followed by biological anaerobic organic acid production through fermentation, which lowers the feedstock pH and effectively preserves biomass during storage (McDonald et al., 1991). Significant attention has been given to ensiling for preservation of forage for livestock feed with specific focus on the requirements that initiate oxygen exclusion, additives including mineral and organic acids to exclude unwanted microbial activity, low-cost sugars to encourage growth of fermentative organisms, and microbial inoculants that produce well-preserved silage (Yitbarek and Tamir, 2014). Ensiling of winter crops has recently received attention for its potential to provide a year-round source of biomass for on-farm anaerobic digesters to provide renewable electricity (Dale et al., 2016).

The preservation of corn stover through ensiling as a bioenergy feedstock has been evaluated in the laboratory (Richard et al., 2001; Chen et al., 2012; Liu et al., 2013), although field-scale trials have been limited to livestock forage applications (Shinners et al., 2007, 2011). However, corn stover is typically harvested at lower initial moisture content compared to feedstock dedicated for forage and, thus, contains increased interstitial oxygen that must be removed or biologically consumed in order to establish fermentation conditions. Despite this challenge, low-moisture ensiling at 40–50% moisture (wb) has successfully preserved dry matter in corn stover for >6 months (Shinners et al., 2011) and remains a potential solution for stabilizing corn stover considered too wet for stable dry bale storage.

The “Ritter” pile was designed for the preservation of wet fiber and digestion of the non-fiber component into pulp and/or paper (Ritter, 1960). The Ritter pile, which has been applied to bagasse (Morgan et al., 1974; Kruger et al., 1981) and corn stover (Hettenhaus et al., 2000; Atchison and Hettenhaus, 2003), is constructed and compacted using a slurried biomass; water is recirculated continuously in the traditional Ritter pile and only during pile formation in the modified Ritter approach (Atchison and Hettenhaus, 2003). The pile remains saturated at 70–80% moisture (wb) for the duration of storage except when pretreatment by acid impregnation of the biomass is necessary (Humbird et al., 2011), whereupon a dewatering step is included. Fortunately, the dewatering stream contains nutrients that could be applied to cropland as irrigation water to offset fertilizer application (Hettenhaus et al., 2000). One disadvantage to the Ritter method is that it is very water intensive and requires extensive infrastructure for the slurrying and recirculating process, increasing the cost and reducing environmental sustainability.

Two anaerobic wet storage methods, ensiling and the modified-Ritter storage system, were evaluated in this study because they have tremendous potential to secure corn stover from losses due to fire or microbial degradation, which are both significant vulnerabilities of dry storage systems at industrially relevant scales. These storage studies were necessary to evaluate the applicability of ensiled storage for biochemical conversion pathways, where cellulose and hemicellulose in biomass are depolymerized to monomeric carbohydrates through a combination of pretreatment and enzymatic saccharification followed by fermentation to fuels and/or chemicals. Unfortunately, the majority of research on ensiling assesses carbohydrate composition in storage performance using forage industry-specific terms, which are not directly correlated to fuel yield at a biorefinery (Wolfrum et al., 2009). To better understand this response, laboratory-based storage studies were conducted to characterize the performance of each storage method as related to as-harvested material, followed by dilute acid or alkali pretreatment and subsequent enzymatic saccharification to sugar monomers. The laboratory-based storage studies were also performed under high-moisture aerobic conditions to determine the potential impact of aerobic degradation in storage. Finally, a field-scale study was conducted to demonstrate the effectiveness of the wet logistic system using ensiling.

## Materials and Methods

### Storage Reactor and Field Experimentation

Laboratory-based anaerobic storage experiments were conducted to simulate ensiling in a drive-over pile and a modified-Ritter pile. For these experiments, Pioneer P1151 corn stover was harvested in September of 2014 in Stevens County, KS following the grain harvest using a forage harvester, and then *Lactobacillus plantarum* was applied to the stover prior to collection in a forage wagon. Stover was immediately compacted in 55 gal drums at an initial moisture content of 47% (wb), sent to Idaho Falls, ID in a refrigerated semi-trailer, and then stored at −20°C at a local food storage facility until use. For ensiling, duplicate 100 L reactors were each loaded with 5.9 ± 0.5 kg corn stover dry basis (db) at an initial moisture content of 47.3 ± 1.5% (wb; *n* = 4). To simulate the modified-Ritter pile formation, slurrying was performed by washing 22.5 kg stover (db) with the equivalent of 6.2 kg water per kg dry stover continuously for 1 h prior to loading duplicate reactors with 6.4 ± 0.5 kg stover (db) at 76.0 ± 0.6% (wb; *n* = 4) moisture. For both anaerobic storage methods, a pressure of 3.9 kPa was applied to the biomass at five separate 300 s intervals during loading in order to maintain homogeneity between reactors and a final packing density of 73.7 and 76.9 kg/m^3^ (db) for the ensiling and modified-Ritter reactors, respectively. Storage reactors were modified from a previous design (Wendt et al., 2014; Bonner et al., 2015) to maintain anaerobic conditions and equipped with an aluminized gas sampling bag to capture and quantify gas formation during the 110-day storage period. Moisture content and gravimetric DML were calculated based on randomly selected samples, as described previously (Wendt et al., 2014).

**Figure 1:**
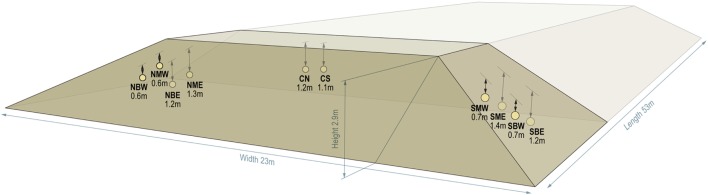
Field storage pile and locations of monitoring points for temperature, gas formation, and dry matter loss. Yellow dots indicate monitoring locations at various depths and pile locations. Monitoring locations are abbreviated as follows: N = north, S = south, E = east, W = west, B = bottom, M = middle, C = center.

An outdoor storage pile experiment was performed concurrently with aerobic laboratory experiments using corn stover, Pioneer P1151AMX, which was harvested in Stevens County, KS in August 2015. The corn stover was harvested at an initial moisture content of 40.5 ± 1.6% (wb; *n* = 4) using a windrowing flail-shredder (Hiniker model 5630HL, Mankato, MN, USA) followed by a forage chopper (John Deere model 7780, Moline, IL, USA) with a windrow pickup head (John Deere model 640°C, Moline, IL, USA). The stover was blown into a self-unloading silage truck wagon (Taller Fehr 11.5 m silage trailer, Cuauhtémoc Chihuahua, Mexico) and transported to a nearby storage site where a 272 t (db) pile was formed. Pile dimensions were 23 m wide, 3.5 m tall, and 53 m long; the pile was shaped and compacted using a tractor (John Deere model 9420, Moline, IL, USA). A longitudinal section of the pile was selected for real-time monitoring based on solar irradiation and spatial location, with specific locations shown in Figure 1. Gas sampling ports, temperature probes (Campbell Scientific, Logan, UT, USA), and “DML bags” were placed in 10 locations within this section of the pile 1 day after pile formation to monitor storage performance. DML bags consisted of 10 cm diameter Drain-Sleeve^®^ filter fabric (Clariff Corporation, Midland, NC, USA) cut into 45 cm pieces, filled with corn stover obtained from the pile and secured with zip-ties at the ends. Hourly temperature measurements were recorded for the external air at each of the 10 zones and at an additional surface location on top of the pile. The pile was left initially undisturbed for about 3 weeks prior to covering with plastic wrap, which was later added to limit additional air infiltration. Interstitial gas evolution was sampled at 7 points during the storage period, and gas was transferred into aluminized bags following purging each sampling line with five volumes. After 6 months of storage, the pile was sampled for DML, pH, organic acids, moisture content, composition, and pretreatment and enzymatic hydrolysis. DML bags and moisture samples from each of the 10 zones were collected as well as a 200 kg sample from a section of the pile approximately 20 m east of the sampling zone, and this sample is available for public use through the INL Bioenergy Feedstock Library at https://bioenergylibrary.inl.gov.

The 2015 corn stover was shipped overnight to INL and stored aerobically for 111 days in laboratory reactors to simulate the outer regions of an uncovered pile. For the aerobic laboratory storage experiment, two air flow rates were evaluated in duplicate 100 L reactors as described previously (Wendt et al., 2014). Filtered room air was supplied to the reactors at a rate of 0.5 or 1.0 L/min, corresponding to complete air exchange every 200 or 100 min, respectively. For a 0.5 L/min airflow rate, 8.46 and 7.93 kg corn stover (db) were added with the compaction described above to obtain a packing density of 101.41 and 95.10 kg/m^3^ (db), respectively. For an airflow of 1 L/min, 8.35 and 7.37 kg corn stover (db) additions resulted in 100.10 and 88.37 kg/m^3^ packing density, respectively.

### Chemical Compositional Analysis

To test for microbially generated greenhouse gases or air pollutants, the following were analyzed: CO_2_, CH_4_, H_2_, N_2_O, CO, NO_x_. Gas exiting the storage reactors was analyzed using an automated gas chromatograph (MicroGC 3000, Agilent, Santa Clara, CA, USA) as described previously (Wendt et al., 2017). Nitrous gases were measured using Nitrous Gases 100/c and Nitrogen Dioxide 0.5/c Draeger tubes (Draeger Safety Inc., Pittsburgh, PA, USA). Organic acids were extracted from samples prior to and after storage and measured *via* high-performance liquid chromatography (HPLC) according to a method described previously (Wendt et al., 2017).

Chemical compositional analysis was determined for duplicate corn stover composite samples (prior to and after storage) according to standard biomass procedures developed by the National Renewable Energy Laboratory (NREL) (National Renewable Energy Laboratory, 2013), similar to what is reported elsewhere (Wolfrum et al., 2017). Extractives from water and ethanol were produced using an ASE 350 (Dionex, Sunnyvale, CA, USA). Following extraction, the biomass was subjected to a two-stage acid hydrolysis. The liquor from the extraction and subsequent acid hydrolysis was analyzed using HPLC with a refractive index detector (Agilent, Santa Clara, CA, USA). Monomeric sugars were analyzed with an Aminex HPX 87 P column (Bio-Rad, 300 × 7.8 mm, Hercules, CA, USA). Organic acids were analyzed with an Aminex HPX 87 H ion exclusion column (Bio-Rad, 300 mm × 7.8 mm, Hercules, CA, USA). Results for soluble and structural sugars from duplicate analysis runs were normalized to 100% recovery for a NIST Reference 8491 Bagasse sample that was analyzed alongside the samples.

To determine the yields of sugars available in biomass, sugar release was measured following sample pretreatment with dilute acid/dilute alkaline and subsequent enzymatic hydrolysis. Samples analyzed include as-harvested stover from 2014 and 2015, the ensiled and 1 L/min samples, and the field composite. Soluble and structural sugars were considered in the analysis of sugar yields. Dilute acid pretreatment was performed with a Dionex ASE 350 Accelerated Solvent Extractor (Dionex Corporation, Sunnyvale, CA, USA) at 10% (w/w) solids loading by adding 30 mL of 1% sulfuric acid (w/w) in 66-mL Dionium cells, using a method similar to that described by Wolfrum et al. (2013) with a 360 s ramp in temperature to 160°C followed by a 420 s incubation (severity factor = 2.61). The pretreatment liquor and rinse liquid were measured for monomeric and polymeric sugars as well as degradation products, as described above.

Dilute alkaline pretreatment was carried out in 25 mL Incoloy^®^ tube reactors (Alloy Metal and Tubes, Houston, TX, USA) with a fluidized sand bath (Omega FSB-4, Stamford, CT, USA) to supply heat. Dry biomass (2 g) was soaked in a prepared sodium hydroxide solution overnight before being loaded into the tube reactors. Total weight per reaction was 20 g with a 10% (w/w) biomass loading and 0.05 g sodium hydroxide per g biomass. After 140°C pretreatment for 1,620 s (severity factor = 2.61), reactions were quenched by immersing tubes into an ice bath. Pretreatment liquor was collected after centrifugation at 3,000 × rpm (1,811 × *g*) for 300 s. Washing of solids was performed using 100 mM citrate buffer (pH 4.8) followed by centrifugation until the solids equilibrated at a pH of 4.8.

Enzymatic hydrolysis of dilute acid-pretreated biomass was performed using 1 g (db) of washed solids at 10% (w/w) solids loading and 50 mM citrate buffer, pH 4.8 in triplicate, similar to the methods described by Wolfrum et al. (2017). However, enzymatic hydrolysis of dilute alkaline-pretreated biomass was performed using the entire washed sample at a 5% (w/w) solids loading. Cellic^®^ Ctec2 and Cellic^®^ Htec2 enzyme complexes (Novozymes^®^, Franklinton, NC, USA) were added at a loading rate at 40 mg protein/g and 4 mg protein/g dry biomass, respectively. Sodium citrate buffer was supplemented with NaN_3_ to a final concentration of 0.02% in the biomass slurry to prevent microbial contamination. Flasks were incubated at 50°C and 200 rpm for 5 days. Carbohydrates released in dilute acid pretreatment were measured using HPLC with the HPX-87P column as described above, and fermentation inhibitors, including acetate, furfural, 5-hydroxymethylfurfural, and levulinic acid were measured using HPLC with the HPX-87H column, as described above. Carbohydrates released in dilute alkaline pretreatment and enzymatic hydrolysis were measured using HPLC and a refractive index detector (Thermo Fisher Scientific, Ultimate 3000, Waltham, MA, USA) equipped with the HPX-87H column described above. At least two parallel samples were used in all analytical determinations, and data were presented as the mean of replicates.

### Particle Size Distribution (PSD)

Particle size distribution was determined for four representative samples of dry corn stover using a Ro-Tap RX-29 (W.S. Tyler, Mentor, OH, USA) with sieve sizes of 6.35, 3.34, 0.84, 0.42, 0.25, 0.177, and 0.149 mm. The weight percent of corn stover on each sieve was calculated after a 600 s vibration operating time (ASAE Standards, 1992; 47th ed.).

### Statistical Analysis

Averages and one SD are presented with *n* = 2 unless otherwise noted. For chemical composition data of samples (two duplicates included per sample), an unequal variance *t*-test was performed in Microsoft Excel to determine significant differences between each stored sample and the corresponding as-harvested source biomass. For sugar release experiments (*n* = 3), single-factor one-way analysis of variance (ANOVA) was performed in SigmaPlot (version 13.0) to identify significant differences, and Tukey’s honest significant difference test was performed for multiple-level comparison of statistical equivalency if the ANOVA was significant at *p* < 0.05.

## Results

To determine the impact of wet storage on biomass, corn stover was treated with two laboratory anaerobic wet storage methods (traditional ensiling and modified-Ritter storage) as well as two laboratory aerobic wet storage scenarios (0.5 and 1.0 L/min supplied airflow) that simulate wet aerobic field storage. Parameters including gas production, fermentation products, fate or loss of the total dry matter, chemical composition of the stored solids, and availability of sugars for fermentation were analyzed for each condition. In addition, a 272 t (db) corn stover drive-over pile was constructed to evaluate ensiling in the field.

### Particle Size Reduction in the Field

**Figure 2:**
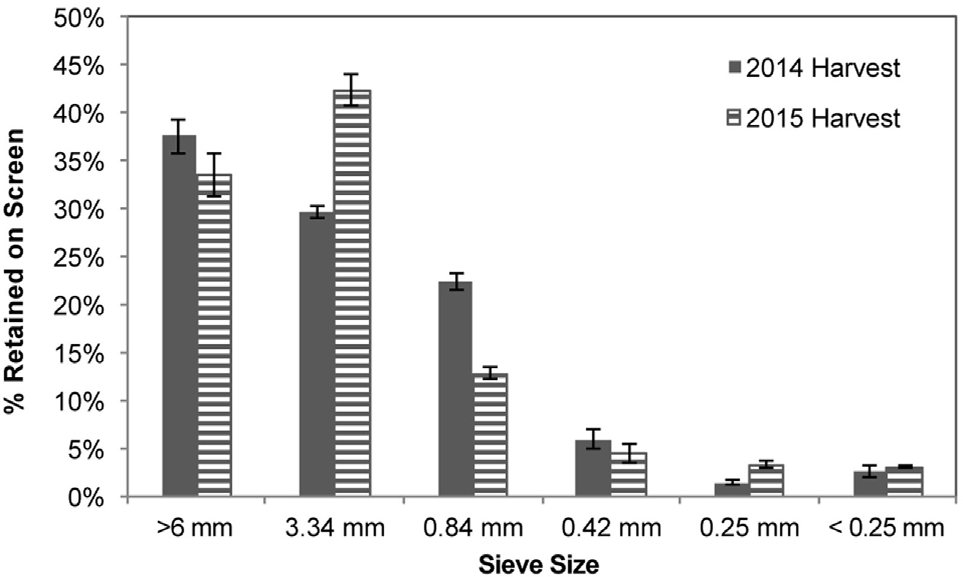
Particle size distribution of forage-chopped corn stover used in laboratory and field storage experiments.

The PSD of the forage-chopped corn stover used in the storage experiments was measured for the two harvest years (Figure 2). In the 2014 harvest, direct cutting and chopping (one-pass harvest) of the standing corn stover left <38% of the stover retained on the 6 mm screen, with the majority of the stover retained on the 3.4 mm (30%) and 0.84 mm (22%) screens and 4.1% below the 0.25 mm screen was considered fines. A slightly different distribution was obtained from the harvesting operation in 2015, where the flail chopping windrower performed initial size reduction followed by a second size reduction in the forage chopping operation (two-pass harvest). A narrower PSD was produced with this harvest approach; 33% of the stover was retained on the 6 mm screen whereas 45 and 13% was retained on the 3.4 and 0.84 mm screens, respectively, and 6.5% of material considered fines. The geometric mean particle size was 4.73 ± 8.19 mm in the 2014 harvest compared to 4.81 ± 6.62 mm in the 2015 harvest.

### Storage Biomass Gas production, Temperature, and Mass loss

CO_2_ released from biomass during storage can be linked to microbial degradation of sugars due to aerobic microbial respiration or to a lesser extent as a result of anaerobic fermentation (McGechan, 1990). After 110 days in storage, the modified-Ritter storage produced about three times more CO_2_ relative to ensiling (Table 1) that coincided with higher DML (9.89% modified-Ritter vs 5.75% silage; Table 2). Anoxic conditions were reached in the modified-Ritter method within 7 days, while 1% oxygen was still present after 80 days in samples following the traditional ensiling approach for storage. Permanent gases other than N_2_, O_2_, and CO_2_ (CO, H_2_, NO_X_, NO_2_, N_2_O, and CH_4_) produced throughout the duration of the experiments were measured for each of the anaerobic conditions (Table 1). CH_4_ and N_2_O were not detected in either the ensiled or modified-Ritter reactor storage method. NO_2_ was present in the modified-Ritter reactors at <0.1 ppm and was not detected in the ensiling reactors. In addition, the modified-Ritter reactors produced threefold to fivefold more CO and H_2_ than the traditional ensiling material although both gases were in the low ppm range.

**Table 1:** Gas production in laboratory reactors after 110 days (anaerobic) or 111 days (aerobic) in storage.

	Gas production (mg/kg biomass)				
Sample description	CO_2_	CO	H_2_	NO_X_	NO_2_
Ensiled	6.44 × 10^3^ (0.55)	3.92 (0.41)	2.78 (0.14)	96.40 × 10^−3^ (0)	0 (0)
Modified-Ritter	19.27 × 10^3^ (1.05)	14.89 (0.03)	14.19 (0.05)	482.00 × 10^−3^ (0)	65.00 × 10^−3^ (0)
Aerobic, 0.5 L/min	161.09 × 10^3^ (19.83 × 10^3^)	0	0	BD	BD
Aerobic, 1 L/min	366.53 × 10^3^ (88.43 × 10^3^)	0	0	BD	BD

*Values represent means with one SD in parentheses*.

*BD, below detection*.

**Table 2:** Composition analysis including percent dry matter loss (DML) and organic acid production for corn stover after anaerobic storage by ensiling and the modified-Ritter method as well as field and aerobic storage.

			Organic acids (% of biomass)					
Sample description	DML (%)	pH	Lactic	Acetic	Butyric	Propionic	Succinic	Formic
Ensiled	5.75 (1.04)	4.62 (0.04)	3.1 (0.07)	0.86 (0.07)	BD	0.78 (0.10)	0.54 (0.02)	0.003 (0.00)
Modified-Ritter	9.89 (0.61)	5.18 (0.03)	BD	5.41 (0.02)	1.45 (0.02)	0.09 (0.02)	0.02 (0.002)	BD
Aerobic, 0.5 L/min	32.10 (1.54)	5.91 (0.04)	0.58 (0.04)	0.86 (0.05)	0.87 (0.06)	2.10 (0.14)	1.73 (0.11)	0.07 (0.005)
Aerobic, 1 L/min	29.52 (5.33)	6.46 (0.43)	0.41 (0.13)	0.80 (0.05)	0.41 (0.41)	1.10 (0.89)	0.96 (0.61)	0.05 (0.02)
Field stored	4.36 (3.25)	4.92 (0.17)	0.51 (0.26)	0.46 (0.05)	0.34 (0.14)	1.14 (0.24)	0.79 (0.24)	0.28 (0.06)

*Values represent means with one standard deviation in parentheses. Not shown are organic acids (valeric, 2-methylbutyric, isobutyric, and isovaleric acid) below detection levels in the samples*.

*DML, dry matter loss; BD, below detection*.

**Figure 3:**
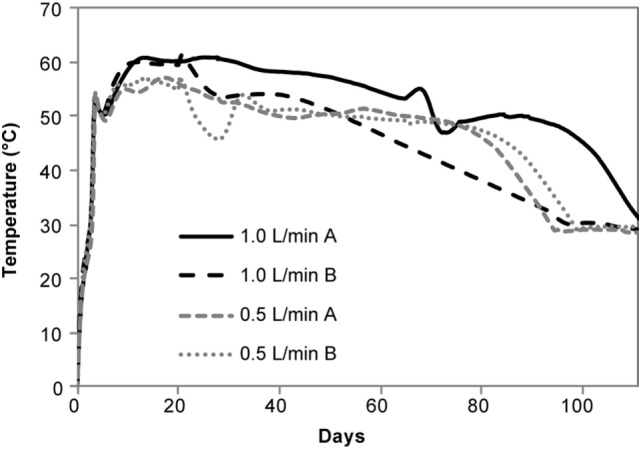
Temperature profile of chopped stover in aerobic reactors.

CO_2_ production ranged from 161 to 366 g/kg in the aerobic reactors and DML varied from 32.10 ± 1.54% to 29.52 ± 5.33% for the 0.5 and 1 L/min conditions, respectively. The other permanent gases listed in Table 1 were not detected in the aerobically stored corn stover in the laboratory reactors due to the dilution of these gases with air flow through the reactors. Significant self-heating occurred in the aerobic reactors through the duration of storage (Figure 3) as a result of microbial respiration of the available carbohydrates. Temperatures peaked at 60°C in 1 L/min reactors within the first 10 days of storage, whereas the 0.5 L/min reactors reached only 57°C. Despite maximum temperatures being only 3°C different, CO_2_ production in the 1 L/min reactors over the first 10 days was over twice that of the 0.5 L/min condition (data not shown). For the duration of the experiments, the aerobic laboratory reactors generally remained at >50°C, whereas the anaerobic laboratory reactors never increased above ambient room temperature. The replicate in the 1 L/min condition with lower packing density exhibited a 5°C drop in temperature after 20 days in storage and cooled sooner than the replicate with higher packing density, corresponding to a lower dry matter loss rate (24.2 vs 34.9%) and contributing to the high SD in DML for the 1 L/min condition.

**Figure 4:**
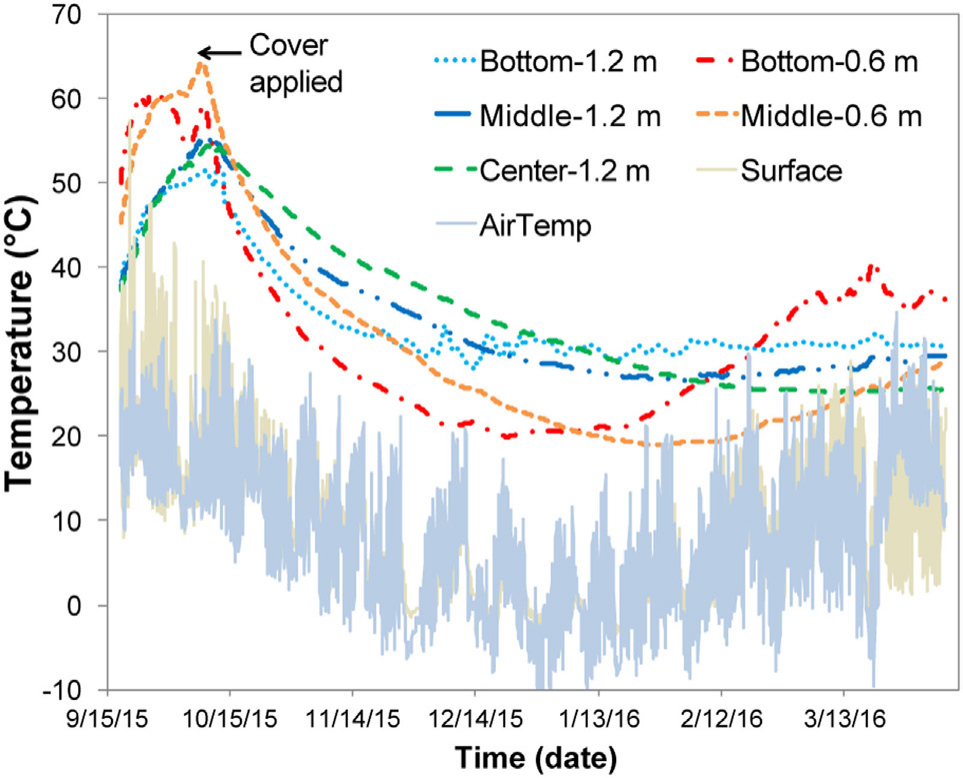
Temperature profile of chopped stover field pile. Each zone is an average of the north and south zones.

For the field experiment, the pile exhibited self-heating during the initial 3 weeks (Figure 4) with temperatures reaching 60°C and decreasing to 20–30°C upon covering the pile with a tarp. Covering the pile was accompanied by a reduction in oxygen levels to <3% and an increase in CO_2_ to levels of 18–22% (data not shown); variability was assumed to result from the inhomogeneous nature of the silage pile relative to the laboratory reactors. For the field samples, up to 400 ppm CO was detected initially, and CH_4_ (~200–300 ppm) was detected by the end of the experiment (data not shown). The field-ensiled material had a DML that was comparable to the laboratory anaerobic wet storage samples after 6 months of storage (4.36 ± 3.25%, *n* = 10) despite the self-heating period that occurred during the first weeks of storage.

### Fermentation Organic Acids and pH

Organic acid concentrations and pH were measured in samples collected at the end of the storage period to characterize fermentation products (Table 2). The ensiled corn stover had a pH of 4.62, lower than that observed for the other storage methods tested (modified-Ritter, pH = 5.18; aerobic, pH = 5.91 and 6.46, 0.5 L/min and 1 L/min, respectively) and contained the highest level of lactic acid (3.10%). Samples from the field-stored silage pile had a similar composite pH (pH = 4.9, *n* = 10) to the ensiled material and lactic acid concentrations near 0.5%, however butyric acid (0.36%) was also present in many samples. Acetic acid (5.41%) and butyric acid (1.45%) were the primary fermentation products of the stover stored in the modified-Ritter system. Propionic (2.10%) and succinic acid (1.73%) were produced in the 0.5 L/min condition, whereas these levels were reduced by nearly half in the 1 L/min scenario.

### Ash, Structural, and Soluble Sugars

Chemical analyses were performed on samples from each storage experiment alongside the respective starting material (as-harvested) to determine the effect of storage on composition (Tables 3 and 4). Total ash is defined as the combination of structural, or physiological, ash and the extractable inorganics fraction that typically contains exogenous soil entrained during harvest and collection operations. Total ash was 19.4% in 2015 samples that made contact with the ground during the two-step harvest method as compared to 9.0% in the direct-harvested 2014 samples, resulting in a proportional decrease in compositional components (i.e. carbohydrates, lignin, and protein) relative to the total stover mass. Washing of the corn stover from the 2014 harvest to stimulate the modified-Ritter storage reduced the extractable ash content from 5.8 to 3.5% and total extractives from 22.3 to 15.6%, meanwhile proportionally increasing the structural components, including the sugars and lignin. In the anaerobic storage experiments, both ensiling and modified-Ritter storage methods resulted in significant decreases in soluble glucan and xylan as well as total extractives. Other than protein levels and soluble galactose (ensiled only), no other statistically significant differences were observed as a result of ensiling or modified-Ritter storage.

**Table 3:** Percent chemical composition analysis of corn stover stored under conditions that were anaerobic, aerobic, and in the field.

	Total ash		Protein	Total extractives	Total carbohydrates				Lignin	Acetate
Sample description	Structural	Extractable			Glucan	Xylan	Galactan	Arabinan		
2014, As-harvested	3.2 (0.4)	5.8 (0.6)	4.5 (0.1)	22.3 (0.2)	33.7 (0.9)	15.9 (0.0)	1.5 (0.4)	2.4 (0.5)	14.6 (0.3)	2.6 (0.3)
Ensiled, Lab	4.0 (0.4)	6.3 (0.8)	3.9 (0.3)	21.0 (0.4)	33.2 (0.8)	16.2 (0.4)	1.4 (0.2)	2.4 (0.3)	14.6 (0.2)	2.6 (0.3)
*p*-Value	*0.122*	*0.527*	*0.042*	***0.011***	*0.609*	*0.160*	*0.815*	*0.992*	*0.913*	*0.894*

2014, Washed	4.6 (0.3)	3.5 (0.4)	3.7 (0.3)	15.6 (0.2)	34.9 (0.8)	16.6 (0.5)	1.6 (0.2)	2.8 (0.6)	15.3 (0.4)	2.6 (0.1)
Modified-Ritter, Lab	4.9 (0.6)	3.5 (1.0)	2.7 (0.1)	12.9 (0.1)	35.4 (0.3)	17.1 (0.2)	1.4 (0.1)	2.4 (0.4)	15.9 (0.2)	2.9 (0.3)
*p*-Value	*0.499*	*0.962*	*0.129*	***0.034***	*0.557*	*0.415*	*0.389*	*0.612*	*0.279*	*0.214*

2015, As-harvested	8.5 (0.8)	10.9 (1.6)	3.8 (0.4)	28.7 (1.7)	31.5 (0.9)	14.9 (0.4)	1.5 (0.0)	2.9 (0.0)	11.3 (0.4)	2.3 (0.1)
Aerobic, 0.5 L/min	9.2 (1.3)	5.8 (0.5)	4.3 (0.0)	19.2 (0.5)	31.1 (1.2)	16.8 (0.4)	1.7 (0.1)	3.2 (0.1)	15.1 (0.3)	1.3 (0.0)
*p*-Value	*0.448*	***0.022***	*0.185*	***0.008***	*0.653*	***0.003***	***0.006***	***0.009***	**<*0.001***	**<*0.001***
Aerobic, 1 L/min	7.8 (0.7)	6.4 (0.2)	4.2 (0.2)	33.5 (1.9)	28.9 (0.5)	15.0 (0.3)	1.5 (0.2)	2.6 (0.4)	13.9 (0.6)	1.1 (0.3)
*p*-Value	*0.309*	***0.034***	*0.242*	***0.019***	***0.022***	*0.743*	*0.582*	*0.251*	**<*0.001***	***0.004***
Field stored	8.2 (1.6)	6.1 (0.9)	4.5 (0.4)	32.3 (2.0)	28.3 (1.1)	14.4 (0.5)	1.3 (0.2)	2.8 (0.2)	11.8 (0.5)	1.7 (0.1)
*p*-Value	*0.672*	***0.025***	*0.087*	***0.040***	***0.009***	*0.170*	***0.001***	***0.027***	*0.122*	**<*0.001***

*Samples were collected for analysis before (as-harvested) and after storage experiments. Values represent means with one SD in parentheses. *p*-Values (italicized) in bold are <0.05 and considered statistically significant as measured by an unequal variance *t*-test comparing stored samples to corresponding performed on as-harvested material*.

**Table 4:** Analysis of percentage of corn stover sugars stored under conditions that were anaerobic, aerobic and in the field.

	Soluble sugars				Structural sugars			
Sample description	Glucan	Xylan	Galactan	Arabinan	Glucan	Xylan	Galactan	Arabinan
2014, As-harvested	1.6 (0.1)	0.3 (0.0)	0.3 (0.0)	0.3 (0.0)	32.1 (1.0)	15.6 (0.0)	1.2 (0.4)	2.2 (0.5)
Ensiled, Lab	0.8 (0.0)	0.2 (0.0)	0.2 (0.0)	0.2 (0.0)	32.4 (0.9)	16.0 (0.4)	1.2 (0.2)	2.2 (0.4)
*p*-Value	***0.007***	***0.026***	***0.012***	*0.575*	*0.749*	*0.096*	*0.877*	*0.962*

2014, Washed	1.0 (0.0)	0.2 (0.0)	0.2 (0.0)	0.2 (0.1)	33.9 (0.8)	16.4 (0.5)	1.4 (0.2)	2.6 (0.7)
Modified-Ritter, Lab	0.4 (0.0)	0.1 (0.0)	0.2 (0.0)	0.1 (0.0)	35.0 (0.3)	17.0 (0.2)	1.2 (0.1)	2.4 (0.5)
*p*-Value	***0.002***	***0.002***	*0.114*	*0.269*	*0.276*	*0.344*	*0.451*	*0.763*

2015, As-harvested	6.4 (1.0)	0.3 (0.2)	0.3 (0.1)	0.2 (0.1)	25.1 (1.0)	14.6 (0.2)	1.2 (0.0)	2.7 (0.0)
Aerobic, 0.5 L/min	1.1 (0.0)	0.8 (0.0)	0.4 (0.0)	0.5 (0.0)	30.0 (1.2)	15.9 (0.4)	1.3 (0.1)	2.8 (0.1)
*p*-Value	***0.011***	*0.059*	*0.087*	***0.012***	***0.002***	***0.002***	*0.101*	*0.330*
Aerobic, 1 L/min	3.3 (0.6)	3.2 (1.1)	0.7 (0.0)	1.1 (0.0)	25.6 (0.4)	11.8 (1.4)	0.8 (0.2)	1.5 (0.4)
*p*-Value	***0.013***	***0.010***	***0.005***	***0.001***	*0.457*	***0.024***	***0.018***	***0.010***
Field stored	4.5 (0.7)	0.6 (0.3)	0.5 (0.1)	0.6 (0.1)	23.8 (1.3)	13.9 (0.6)	0.8 (0.2)	2.2 (0.2)
*p*-Value	*0.060*	*0.198*	***0.013***	**<*0.001***	*0.121*	***0.011***	**<*0.001***	**<*0.001***

*Samples were collected before (as-harvested) and after storage experiments. Values represent means with one SD in parentheses. *p*-Values (italicized) in bold are <0.05 and considered statistically significant as measured by an unequal variance *t*-test comparing stored samples to corresponding performed on as-harvested material*.

Aerobic storage in the two laboratory conditions resulted in many statistically significant changes compared to the as-harvested material; extractable inorganics decreased, acetate decreased, and lignin was enriched. Total extractives decreased from 28.7 to 19.2% in the 0.5 L/min scenario but increased to 33.5% in the 1 L/min case. Additional changes were observed in the carbohydrates in terms of soluble, structural, and total levels. For the 0.5 L/min scenario, structural glucan was enriched from 25.1 to 30.0% with a corresponding decrease in soluble glucan from 6.4 to 1.1%, resulting in no overall difference in total glucan. Total xylan, galactan, and arabinan were all significantly enriched as a result of this lower airflow condition, although only the increase in structural xylan and soluble arabinan was significant. For the 1 L/min scenario, total glucan was reduced from 31.5 to 28.9% due to the reduction of soluble glucan from 6.4 to 3.3%. Soluble xylan, galactan, and arabinan all increased significantly spurred by a decrease in the corresponding structural counterparts; however, the total levels were unchanged despite the fact that solubilization occurred.

Field-stored samples were taken from multiple zones in the pile and data were combined for simplicity (*n* = 6 zones). Many of the compositional changes observed in the field generally followed the trends of the 1 L/min condition, with a significant reduction of extractable ash, increase in total extractives, and reduction of total glucan compared to the as-harvested material and no significant difference compared to the 1 L/min condition (*p* > 0.05, not shown). Significant loss of acetate and structural xylan, galactan, and arabinan was also evident in the field sample. Notable differences were observed in field samples stored at 0.6–0.7 m depths compared to 1.2–1.3 m depths. For example, structural glucan was reduced from 25.1 to 22.6% at the 0.6–0.7 m depth (*p* = 0.025, *n* = 2) and structural xylan was reduced from 14.6 to 13.3% (*p* = 0.002, *n* = 2), but there was no significant difference in either component at 1.2–1.3 m depths with glucan at 24.4% (*p* = 0.34, *n* = 4) and xylan at 14.2% (*p* = 0.100, *n* = 4). Acetate was also reduced from 2.3% at the time of harvest to 1.6% at the 0.6–0.7 m depth (*p* = 0.003, *n* = 2) but only to 1.8% at the 1.2–1.3 m depth (*p* = 0.003, *n* = 4).

### Pretreatment and Enzymatic Hydrolysis

**Figure 5:**
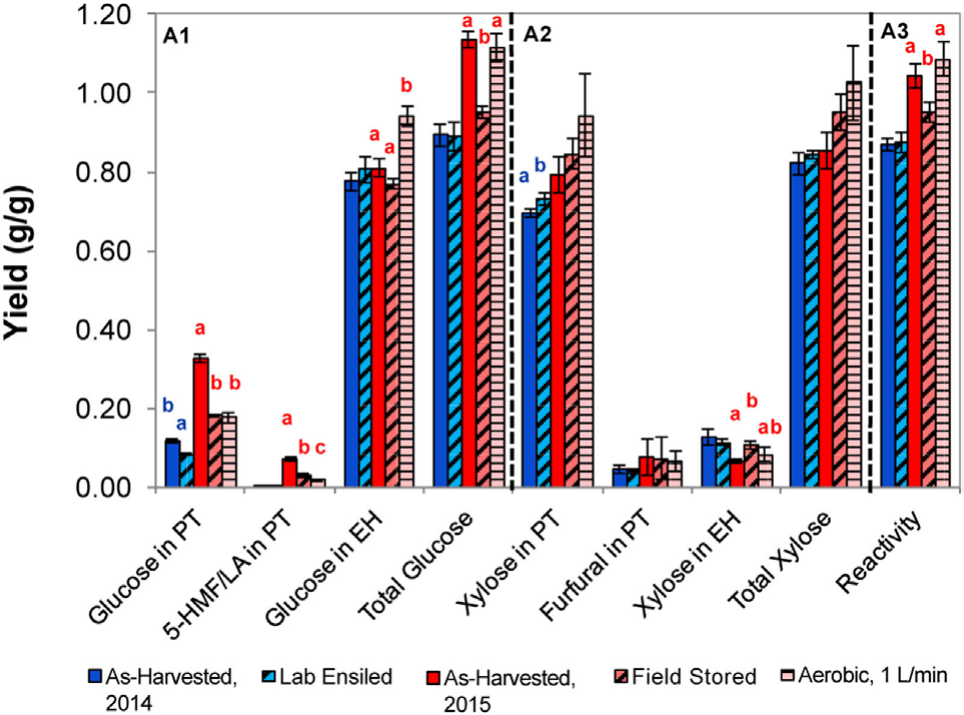
Sugars released from corn stover with dilute acid pretreatment and enzymatic hydrolysis for the as-harvested or stored samples (A1, glucose; A2, xylose; A3, reactivity). Error bars represent the standards of deviation (*n* = 3). Letters represent statistically distinct values as determined by Tukey’s test.

**Figure 6:**
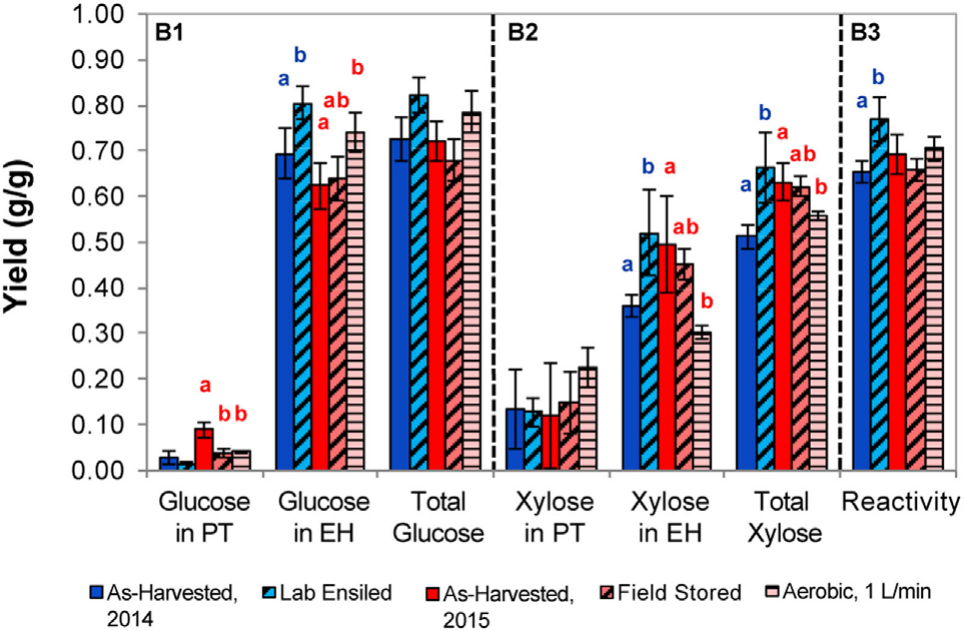
Sugars released from corn stover with dilute alkaline pretreatment and enzymatic hydrolysis for the as-harvested or stored samples (B1, glucose; B2, xylose; B3, reactivity). Error bars represent the standards of deviation (*n* = 3). Letters represent statistically distinct values as determined by Tukey’s test.

The laboratory-ensiled corn stover, field-stored stover, and the stover from 1 L/min airflow laboratory condition, along with their as-harvested counterpart, were subject to biomass pretreatments using dilute acid or dilute alkali, both followed by enzymatic hydrolysis for determining total sugar release. Figures 5 and 6 show the proportion of glucose, xylose, and fermentation inhibitors as a result of pretreatment and enzymatic hydrolysis. Total reactivity is also presented, which is a measure of total glucose and xylose yield from the total structural and non-structural carbohydrates in the corn stover.

Laboratory-ensiled corn stover from the 2014 harvest had minor yet statistically significant variations compared to as-harvested stover as a result of dilute acid pretreatment. For example, glucose was slightly higher for the as-harvested sample, but xylose yield following acid pretreatment was increased as a result of laboratory-ensiled storage. No changes were seen in subsequent enzymatic hydrolysis of these two samples, resulting in the net effect of no statistically measurable difference in feedstock reactivity as a result of laboratory ensiling with the dilute acid pretreatment approach. Within the 2015 samples, significant differences were observed as a result of dilute acid pretreatment and enzymatic hydrolysis. Stored samples released approximately half of the glucose after pretreatment compared to the as-harvested stover, yet increased glucose was released in enzymatic hydrolysis in the aerobic storage condition. This resulted in similar total glucose yields for the as-harvested and aerobically stored samples, significantly higher than the field-stored material, and this trend was conserved in final feedstock reactivity measurements.

An evaluation of the samples collected from the 2015 harvest showed markedly different response to dilute acid pretreatment. First, there was higher total soluble glucose released and ultimately higher reactivity as compared to the 2014 season samples; however, this was not surprising considering that the soluble glucan levels in the 2015 as-harvested field samples were also several fold greater than the 2014 as-harvested samples (5.9 vs 1.0–1.6%, respectively, Table 4). However, the 2015 samples produced significantly more fermentation inhibitors than in 2014. In the as-harvested samples, soluble glucose was degraded to 5-HMF and levulinic acid (0.071 g inhibitor/g dry biomass), whereas in the field-ensiled sample less glucose was converted to 5-HMF (0.031 g inhibitor/g dry biomass) without levulinic acid formation (data shown represent the sum of 5-HMF and levulinic acid). By contrast, less than 0.004 g/g 5-HMF was produced from glucose degradation for the laboratory-ensiled samples (2014 harvest) and levulinic acid was not produced.

Dilute alkaline pretreatment and enzymatic hydrolysis results are presented in Figure 6. In the laboratory-ensiled samples, no difference in sugar release in pretreatment was observed compared to the as-harvested samples but total glucose and xylose release in enzymatic hydrolysis were significantly increased, resulting in higher feedstock reactivity. For the field-ensiled samples, there was a statistically significant reduction in glucose release after alkaline pretreatment compared to the as-harvested material, which is consistent with the dilute acid pretreatment. However, no other significant difference in sugar yield was measured due to field ensiling, resulting in no significant difference in feedstock reactivity in the field-stored vs as-harvested sample. Similar to the 2014 samples, aerobic storage led to increased glucose yield in enzymatic hydrolysis; however, a significant reduction of xylose release in enzymatic hydrolysis resulted in a final material that had a similar reactivity to the as-harvested stover.

## Discussion

### Particle Size Reduction in the Field

Forage chopping is a common practice employed when making silage for livestock feed, and the size reduction serves to improve packing density and, thus, limit oxygen infiltration during storage. Likewise, forage chopping of high-moisture feedstock can be used for biofuels-related crops and can reduce the preprocessing required to meet <6 mm target size specification for a biorefinery (Humbird et al., 2011). For the one- and two-pass corn stover harvesting operations tested, only 38 and 33% of the biomass would need further size reduction to meet size specification, respectively. While the geometric means from the two harvest years are similar and are in the range of previous reports for forage-chopped corn stover (Shinners et al., 2011), the increased SD in the 2014 harvest suggests a wide particle size range compared to the 2015 harvest. Geometric mean is often reported in forage-specific literature, and yet full PSD information is necessary to meet size specifications at a biorefinery and to identify opportunities for fractional removal of on-specification materials. For example, Lisowski et al. (2017) cite the need for stringent PSDs when using biomass for bioenergy purposes and provide PSDs for a range of forage-chopped high-moisture energy crops. For the two-pass system, placing the corn stover on the soil between the flail shredding and forage chopping operations resulted in more soil entrainment in the biomass, as evidenced by an increase in total ash into the system from 9% for the 2014 harvest and 19% for the 2015 harvest (Table 3). Therefore, while the two-pass harvesting operation resulted in better size reduction and would require less preprocessing at a biorefinery to meet target size specification, it also resulted in increased soil (ash) content that would require removal and disposal at the biorefinery.

### Anaerobic Storage Performance

The preservation of biomass during storage is a key indicator in the success of a particular storage approach. Likewise, gas production is an important factor in evaluating a storage system for greenhouse gas and air pollutant potential and thus was measured in the wet storage conditions tested. The increased production of CO_2_ and other permanent greenhouse gasses in the modified-Ritter storage reactors along with higher DML suggested greater microbial activity than for the traditional ensiling material. The presence of CO has been documented in composting of green and municipal solid wastes and has been related to a physio-chemical process that occurs in the presence of oxygen during the initial stages of composting (Hellebrand and Kalk, 2001; Phillip et al., 2011). Hellebrand and Kalk demonstrated elevated CO levels at the beginning of composting and after each aeration episode, while Phillip et al. showed that CO levels were elevated in sterilized compost compared to non-sterilized. For the corn stover experiments, the highest CO levels were measured in the first 6 days of storage in the anaerobic reactors as well as in the first 7 days of the field storage demonstration (data not shown). This further supports the claim that anaerobic storage conditions should be established for high-moisture biomass to prevent both degradation and greenhouse gas production.

Successful ensiling results from the fermentative production of lactic acid from soluble sugars and is carried out by homolactic and heterolactic bacteria that help acidify material and prevent other microorganisms from degrading the dry matter; other compounds, such as acetic acid, may also be produced depending on the predominant fermentation pathways (McGechan, 1990; McDonald et al., 1991). The ensiled material was characterized by significant lactic acid, while the modified-Ritter method was primarily acetic and butyric acid. Similarly, Morgan et al. (1974) and Atchison and Hettenhaus (2003) reported an absence of lactic acid bacteria and lactic acid in sugarcane bagasse stored using the Ritter methods but observed the presence of acetic, butyric and propionic acid, likely due to the presence of *Clostridia* and *Bacillus* species in the feedstock.

In the present study, the lack of lactic acid and presence of butyric acid in the modified-Ritter material suggested that the washing process, used to simulate the slurrying of biomass in the Ritter method, may have had a negative effect on the lactic acid fermentation process. While a lactic acid bacteria inoculant could be added to the modified-Ritter storage method after the simulated slurrying, there is little practicality of doing this during field-scale storage as it would be challenging and costly to execute. The washing step of the as-harvested material was successful in removing about 40% of the soil contamination, which is measured as extractable inorganics. Similarly, total extractives and soluble glucan and xylan were significantly reduced by approximately one third as a result of the washing step. While there are benefits to removing these soluble components, primarily ash, prior to conversion, the additional DML observed in the modified-Ritter storage suggests that washing removed the necessary soluble sugars for successful fermentation. Overall, the organic acid profiles along with the DML and gas production observation suggest that the lactic acid fermentation associated with the ensiling storage method resulted in superior performance compared to the modified-Ritter and aerobic storage methods in the laboratory.

Anaerobic storage conditions preserved not only dry matter compared to aerobic storage but also the primary compositional components including the structural sugars. Similar observations for glucan and xylan preservation have been reported when DML in storage remains at 5% (Liu et al., 2013). A slight reduction in soluble sugar components, primarily soluble glucan, was observed as a result of both anaerobic storage methods. This is expected since consumption of the soluble sugars by fermentative microorganisms is necessary to fuel the production of organic acids for biomass preservation. Overall, the anaerobic conditions had minimal effects on composition and also preserved material significantly, and of the two methods, ensiling is the recommended approach due to the reduced DML.

### Aerobic Storage Performance

Aerobic storage in laboratory reactors resulted in approximately 30% total loss of matter over the 111-day storage period. Significant self-heating was observed in the aerobic conditions likely due to microbial respiration of available carbohydrates, with the higher airflow condition exhibiting slightly higher temperatures and producing over twice the CO_2_ relative to the low-flow condition. Similar temperature profiles have been reported in piled corn stover (Shinners et al., 2011) as well as in laboratory-stored corn stover (Wendt et al., 2014; Bonner et al., 2015) and pine chips (Bonner et al., 2015). Self-heating also occurred during the field demonstration prior to covering. The temperatures closest to the surface (about 0.6 m depth) were >55°C, similar to that of the laboratory 1 L/min airflow reactors (~50–60°C for much of the experimental time). Temperatures at depths of ~1.2 m were approximately 50°C, more similar to the 0.5 L/min air flow reactors (~50°C for the first 80 days). These results suggest that the initial maximum temperatures were related to oxygen availability in the pile. Upon covering the field pile to encourage ensiling, the pile temperatures decreased dramatically to 20–30°C (lower temperatures were closer to the surface) suggesting that aerobic microbial activity was suppressed and that anaerobic conditions were established. While the organic acid profile for the field-stored samples was more similar to the 0.5 L/min aerobic case, limiting oxygen to the pile was effective in limiting overall DML to <5%. Considering the 30% DML losses in the aerobic conditions, the low DML achieved in the field suggest anaerobic conditions were prevalent within the storage pile.

In contrast to the relative stability of biomass components (i.e., carbohydrates, lignin, and inorganic nutrients) observed in the anaerobic storage laboratory experiments, aerobic and field storage resulted in measurable compositional changes. Extractable inorganics decreased in all samples, likely due to the consumption of macronutrients to support microbial respiration. A significant reduction in acetate was observed in all samples, suggesting that a mild form of pretreatment likely occurred. Acetate is one of the first structural components to be released during degradation, as acetyl bonds in the hemicellulose (xylan, galactan, and arabinan) are cleaved by microorganisms attempting to break down hemicellulose and later cellulose (primarily glucan). An assessment of the shift of structural xylan, galactan, and arabinan to soluble forms, along with significant acetate reduction, suggested that hemicellulose depolymerization occurred during aerobic storage with the 1 L/min airflow. A different story is evident as a result of storage at the lower airflow rate of 0.5 L/min. While total glucan was preserved under this condition, >75% of the soluble glucan was lost and a concomitant enrichment of structural glucan occurred. Hemicellulose depolymerization was not evident under low-airflow storage condition, as measured by an enrichment of structural xylan. Overall, the structural sugar profiles suggested that the lower airflow condition preserved the sugars better than in the high airflow condition, which were characterized by higher microbial consumption of the structural sugars as well as mild pretreatment effects. Interestingly, hemicellulose depolymerization was seen in the field-stored samples, with solubilization of galactan and arabinan at all pile depths and significant glucan and xylan solubilization in the shallower samples only (0.6–0.7 m depths). These results suggested that the microbial-mediated self-heating that occurred during the initial 3 weeks of storage was sufficient to produce the mild pretreatment effects seen in the 1 L/min airflow condition in the laboratory.

Lignin content was enriched as a result of aerobic storage, where approximately 30% of dry matter was lost. Lignin content is often proportionally enriched as a result of storage loss due to the biodegradation of cellulose and hemicellulose and the inaccessibility of lignin to microorganisms, and similar results have been reported for aerobically stored corn stover (Athmanathan et al., 2015). Not surprisingly, lignin content was not enriched in the field-stored samples due to the low DML that occurred as a result of the anaerobic conditions created after covering.

### Sugar Release

The pretreatment conditions chosen for this study are industrially relevant conditions and were selected to deliver maximum hydrolysis of structural sugars in cellulose and hemicellulose to monomeric sugars for subsequent fermentation to fuels and/or chemicals. In this study, enzymatic hydrolysis followed either acid or alkaline pretreatments in order to determine total glucose and xylose yield as well as feedstock reactivity. Dilute acid pretreatment resulted in glucose and xylose release several fold higher compared to alkaline pretreatment, however subsequent enzymatic hydrolysis improved depolymerization such that total glucose and xylose releases were only marginally less for the alkaline method. Similar trends in dilute acid and alkaline pretreatment have been reported for corn stover (Duguid et al., 2009) and are a response to the different chemistries; acid based chemistries remove hemicellulose so that the cellulose is assessable to enzymatic depolymerization (Mosier et al., 2005), whereas alkaline methods solubilize lignin and leave hemicellulose less affected (Alvira et al., 2010). A portion of the soluble glucan in the sample sets collected from the 2015 season was over-pretreated in the dilute acid method, resulting in a significant formation of fermentation inhibitors in the as-harvested material. However, both field and aerobic storage were effective in lowering the inhibitor levels.

Laboratory-ensiling resulted in no change in feedstock reactivity or a slight increase in reactivity as a result of pretreatment with dilute acid and dilute alkali, respectively, followed by enzymatic hydrolysis. A similar result has been observed in ensiled sorghum subject to dilute alkali pretreatment (Sambusiti et al., 2012) and ensiled corn stover subjected to steam explosion and enzymatic hydrolysis (Liu et al., 2013). The field-stored sample showed slightly less feedstock reactivity in the dilute acid pretreatment approach, and no changes in reactivity were observed with the dilute alkali approach. While the field sample points to a slightly different trend compared to the laboratory-ensiled corn stover, it can be understood by assessing carbohydrate changes as a result of storage. Whereas the laboratory storage experiment was sealed from the atmosphere immediately upon setup, the field-stored pile self-heated for 3 weeks, resulting in lower soluble sugar levels compared to the as-harvested. Therefore, less sugar was available for release in the subsequent conversion tests. Delayed sealing has been widely reported to reduce forage quality for livestock feed (Henderson and McDonald, 1975), and similar practices of quick sealing upon storage pile formation are recommended for the bioenergy industry. However, this field demonstration showed that delayed pile coverage, which may occur as a result of competing priorities during harvest season, resulted in less than 5% DML and that the remaining material yielded equivalent amounts of glucan and xylan upon pretreatment and enzymatic hydrolysis relative to the unstored starting materials. This indicates that biomass producers and aggregators have an operational window in which to finalize storage pile construction and covering without incurring extensive material losses and quality changes.

Laboratory-based aerobic storage experiments (1 L/min airflow) using the same corn stover that was collected for the field study indicated that these samples were slightly more reactive than the field-stored stover, also evidenced by hemicellulose depolymerization (Table 4). However, when total structural sugar availability and release were considered, regardless of pretreatment chemistry, sugar release from these aerobically stored materials was similar to the as-harvested samples (Wolfrum et al., 2013). Herrmann et al. suggest that DML should be accounted for when considering final energy yield from stored biomass (Herrmann et al., 2011); this adjustment was not made in this study such that it would be possible to assess feedstock reactivity in terms of the vastly different storage histories a biorefinery might encounter. There is still debate as to whether the farmer or the biorefinery will pay for any penalties incurred as a result of DML; some models assume a set price for tonnage harvested whereas others pay only for the biomass delivered to the biorefinery gate. Regardless, the loss of >30% total dry matter and the corresponding reduction of carbohydrates as a result of aerobic storage would be unacceptable for the refinery and should be avoided.

The sugar release results provide strong support for the incorporation of wet stored biomass into commercial biochemical conversion processes. The nearly 100% release of sugars along with inhibitor formation suggests that the severity of the dilute acid pretreatment method was too high to accurately measure storage-related changes in this feedstock. This finding highlights two opportunities for cost reduction in conversion, either through reduced pretreatment severity through lower temperatures or acid levels, or for capturing the water extractives—including soluble carbohydrates—prior to pretreatment and defining a value added product stream for a conversion facility. Wolfrum et al. suggest that lowering severity levels is an effective approach to assessing biomass-related responses in conversion (Wolfrum et al., 2013). Other groups have suggested converting soluble sugars to valuable products during storage, for example to ethanol or volatile organic acids, and extracting them prior to conversion (Henk and Linden, 1996; Hamilton et al., 2016). Results of this study show that fermentable sugar release from wet-stored biomass was equivalent to that of freshly-harvested materials using two different commercially-relevant pretreatment methods. This suggests that anaerobic storage of high-moisture corn stover is a potential near-term solution to manage storage losses in commercial biorefinery logistics operations.

## Conclusion

A primary challenge associated with the dry bale logistics system for providing herbaceous materials for bioenergy is the loss of feedstock in storage due to microbial degradation and potential fires. This study demonstrated that wet anaerobic storage is an active management approach for corn stover to preserve biomass in the supply chain. Storage performance was measured in terms of total DML, compositional analysis (i.e., carbohydrates, organic acids, ash, lignin, etc.), gas production, and the potential for sugar release for conversion to biofuels. Both laboratory and field studies showed that long-term stability can be achieved with little effect on feedstock performance in terms of sugar release. Furthermore, this study confirmed that field-chopping and particle size reduction early in the supply chain removed the bulk logistics system’s dependency on drying corn stover prior to baling and could be used to diminish the biorefinery size reduction requirements; in-field forage chopping was capable of reducing over 60% of the corn stover to a particle size of less than 6 mm. Additional opportunities beyond preservation are also possible with wet storage, for example with directed microbial preprocessing for improved convertibility. In summary, incorporating feedstock supply logistics systems centered around high-moisture biomass and wet anaerobic storage offer the potential for biorefineries to reduce the risks associated dry baled feedstock meanwhile providing a feedstock that is compatible with existing conversion technologies.

## Author Contributions

LW, JM, and WS performed a number of experiments and drafted the manuscript. TR and QN executed the field experimentation. TR, LL, and QH also performed experiments. DR, NS, AR, AH, and QN performed data analysis and revised the manuscript.

## Conflict of Interest Statement

The authors declare that the research was conducted in the absence of any commercial or financial relationships that could be construed as a potential conflict of interest. The reviewer MR and handling Editor declared their shared affiliation.
